# The relationship between emotional intelligence and organizational commitment among nurses working in governmental hospitals in Jordan

**DOI:** 10.1186/s12912-023-01361-2

**Published:** 2023-06-08

**Authors:** Islam Al-Oweidat, Ghada Abu Shosha, Tasneem Abu Baker, Abdulqadir J. Nashwan

**Affiliations:** 1 Faculty of Nursing, Zarqa University, Zarqa, Jordan; 2 Nursing Department, Hamad Medical Corporation, P.O. Box 3050, Doha, Qatar

**Keywords:** Emotional intelligence, Emotional regulation, Jordan, Organizational commitment, Nursing

## Abstract

**Introduction:**

Nurses’ emotions and feelings in response to their environment and their ability to manage their emotions can significantly affect several aspects of their job. In Jordan, studies are still investigating whether emotional intelligence is significantly related to organizational commitment.

**Aim:**

To investigate whether a significant relationship exists between emotional intelligence and organizational commitment among Jordanian nurses working in governmental hospitals in Jordan.

**Methods:**

The study used a descriptive cross-sectional correlational design. A convenience sampling method was used to recruit participants working in governmental hospitals. A total of 200 nurses participated in the study. A participant information sheet developed by the researcher was used to obtain the participants’ socio-demographic characteristics, the Emotional Intelligence Scale (EIS) developed by Schutte and colleagues, and the Organizational Commitment Scale developed by Meyer and Allen were utilized for data collection.

**Results:**

Participants had high levels of emotional intelligence (M, SD = 122.3, 14.0) and moderate levels of organizational commitment (M, SD = 81.6, 15.7). Emotional intelligence had a significant, positive relationship with organizational commitment (r = 0.53, p < 0.01). Male nurses, widowed nurses, and nurses with higher postgraduate qualifications demonstrated significantly higher levels of emotional intelligence and organizational commitment than female nurses, single nurses, and nurses with undergraduate degrees (p < 0.05).

**Conclusion:**

Participants in the current study were highly emotionally intelligent and moderately committed to their organizations. Policies supporting the implementation of interventions to improve organizational commitment and maintain a high level of emotional intelligence should be developed and promoted by nurse managers and hospital administrators, as well as decision-makers should magnet the nurses with postgraduate degrees at clinical sites.

## Introduction

Emotions and feelings are crucial aspects of nurses’ lives and play a significant role in work and employees’ behaviours as well. Nurses see the full spectrum of life, from helping neonates latch to their mother’s breasts for the first time to providing dignity and comfort to the death [1, 2]. Consequently, nurses experience the emotions linked to each human milestone such as happiness when a patient gets discharged from hospital, relief when a patient receives adequate analgesia after surgery, pain when rushed to the emergency department following a traumatic accident, sadness when told of the news that the patient is diagnosed with cancer, helplessness when the doctors cannot offer any cure to a rare condition, and grief when a patient dies in the hospital [3].

Healthcare professionals and nurses in particular, should be able to exercise objectivity and practicality when dealing with their patients and families; to be able to provide safe and quality patient care [4]. Otherwise, nurses risk exposing patients to negative attitudes such as bias, discrimination, transference, and inappropriateness that can significantly impact patient health and experience. The key for nurses is to balance their feelings and emotions against the need to remain objective and fair. Nurses can achieve this delicate balance if they possess an adequate level of emotional intelligence [5].

On the other hand, negative emotions, in particular, can result in dissatisfaction, hopelessness, depression, and lack of motivation. The negative impact of low emotional intelligence is contrary to the virtue of organizational commitment, wherein organizational commitment espouses positive emotions in order for an individual to effectively bond, attach and make the decision to remain with and for the organization [6, 7]. When nurses are burned out, tired, exhausted, and unable to control their emotions when carrying out their duties and responsibilities, they become incapable of finding satisfaction with their jobs, meeting their performance standards, and remaining with their jobs [8].

Organizational commitment is a widely studied concept in management and human resources. It is defined by Cioca and colleagues (2021) in their evidence review as the extent to which employees bond to and attach to their organization [9]. The most commonly accepted model of organizational commitment is that of Meyer and Allen (1991), who proposed a structure made up of three components or dimensions, namely (1) affective commitment, (2) continuance commitment, and (3) normative commitment [10].

Employees who were found to be highly committed to their organizations were more likely to be satisfied with their jobs, more involved and engaged with their employers, demonstrate better performance, have lower rates of absenteeism, have lower rates of intention to leave, and demonstrate better psychological well-being [9]. Thus, policies supporting the implementation of interventions to improve nurses emotional intelligence should be developed and promoted by nurse managers and hospital administrators to enhance nurses organizational commitment.

Firstly, gaps in the literature are evident in studies that looked at emotional intelligence. In a review, Kozlowski and colleagues (2017) found that emotional intelligence can influence clinical decision-making. The review found two themes, namely, (1) the subjective experience of emotion and (2) the application of emotion and cognition in clinical decision-making. The investigators found that nurses often demonstrated emotional responses to pressures from their working environments and the care they provide to their patients. When faced with a heavy patient workload, healthcare professionals tend to respond with anxiety, stress, fear, and uncertainty when under pressure. It is not unusual that the emotions nurses felt were triggered and provoked by emotions felt by their patients and the people that surround them. Emotions triggered by the human experience of their patients were wide-ranging and may include annoyance at one end and empathy at the other. There was also a conscious attempt to exclude emotions when making clinical decisions. Healthcare professionals wanted to remain objective in order to be able to account for a scientific approach to patient issues and concerns [11]. The ability to make sound clinical decisions impacts nurses’ feelings of independence and active participation as reliant healthcare professionals, thus potentially increasing their desire to remain with the organization, hence, improving their commitment to the organization [12].

However, a significant limitation of the review was its inability to identify factors that influenced the EI of nurses [11]. Likewise, Salvarani and colleagues (2019) performed a cross-sectional study involving nurses in three hospitals’ emergency departments. Analysis of the results showed that when nurses did not practice mindfulness, did not demonstrate empathy, and had difficulties regulating their emotions and feelings. The higher their perceived experiences of exhaustion and burnout. In turn, nurses with high burnout levels demonstrated lower levels of personal and work satisfaction [13]. It is important to note that investigators did not aim to identify variables that would have promoted mindfulness, empathy, and emotional regulation.

Obviously, without providing adequate focus on emotional intelligence and organizational commitment of nurses, healthcare organizations run the risk of nurses who are highly susceptible to displaying poor performance, low job satisfaction, high rates of depression, burnout and hopelessness, low desire to remain in their current roles, and high levels of intention to leave [14]. In turn, nurses have the propensity not to meet the standards expected of safe and quality patient care, and it is the patients and families who will be negatively affected by inferior bedside practice [15].

The need for studies that explored the linkage between emotional intelligence and organizational commitment of nurses working in Jordanian healthcare settings represents a significant limitation in the current understanding of these variables. Therefore, this study examines the relationship between the emotional intelligence and organizational commitment of nurses working in governmental hospitals in Jordan.

The study results can help nurse managers explore strategies that can boost nurses’ emotional intelligence and improve the level by which they are committed to their organizations. In addition to that, nurse educators can use the study’s results to impart the significance of being aware of one’s emotions, managing them as they arise, and accessing available resources, tools, and support necessary to improve emotional intelligence. Moreover, nurse educators can use the study’s results to formulate educational workshops and capacity-building activities that can increase the level of organizational commitment among nurses.

Lastly, and most importantly, the results of the study can benefit patients, their families, and other significant people since it is their right to be taken care of not only by nurses who can handle their own emotions but also by nurses who are deeply committed to the values of their organizations that are geared towards providing safe and quality patient care.

The current study answers the following research questions:

- What are the perceptions of emotional intelligence abilities and organizational commitment among Jordanian nurses?
- Is there any statistically significant relationship between emotional intelligence and organizational commitment among Jordanian nurses?

## Conceptual framework

**Fig. 1:**
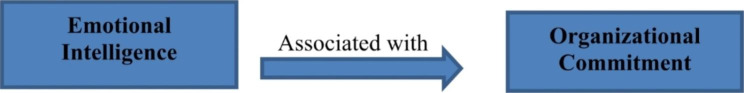
Conceptual framework of the study

The current study was guided by a conceptual framework that was developed by the researchers and based on the literature, in which the independent variable was emotional intelligence and the dependent variable was organizational commitment, whereas the moderators were extracted from the literature as shown in Fig. 1.

## Literature Review

In a previous review, Dugue and colleagues (2021) aimed to examine the current evidence regarding EI and nursing education. Searching through 4 electronic databases and limiting the search to studies published between 2007 and 2021, the investigators identified and included 57 out of 465 articles. The investigators categorized findings into four themes: EI and performance, EI and physical and mental health, EI and social relationships, and EI program. EI was associated with improved productivity, better clinical performance, development of critical thinking skills, and academic success. In addition, better EI can result in better life satisfaction, lower levels of stress and anxiety, and better health and well-being. Regarding social relationships, higher levels of EI correlate with higher levels of empathy, better management of emotion, better listening, better ability to manage conflict, and more significant demonstrations of compassionate care. Lastly, studies highlighted the value of programs in improving EI, which can be via educational or skill interventions [16].

Moreover, following the concept of interventions, Kozlowski and colleagues (2018) performed a quasi-experimental study involving 60 nurses in Australia to test the effectiveness of a brief training intervention in increasing EI levels. The intervention consisted of a workshop, one-on-one feedback session, and individualized follow-up reminder. Results showed that EI levels could be improved by providing a training program [11].

Furthermore, performing an integrative systematic review, Edward and colleagues (2017) examined the concept of emotional intelligence, emotional exhaustion, and emotional labor involving mental health nurses. Theoretically and in practice, mental health nurses’ emotional and mental well-being are at the most risk because of their exposure to patients with pathologic psychological issues and problems. Analyzing results of 20 studies gleaned from searching 4 electronic databases, the investigators found that EI mediates resilience, personal growth, and enhanced emotional management. In turn, higher levels of EI are associated with better nurse retention and less intent to leave. On the other hand, when EI is insufficient, nurses can experience emotional exhaustion, resulting in burnout, stress, and higher rates of staffing attrition. Edward and colleagues (2017) defined emotional labor as the balance between competing levels of EI and emotional exhaustion, therefore recommending that nurses be aware of their emotions and can deal with and manage them [17].

The findings of Edward and colleagues (2017) are those of Park and Park (2020), who performed multiple regression analyses to identify factors that influence turnover intention. The study was participated in by 305 nurses working in emergency departments across South Korea. Findings showed that EI, emotional labor, and social support were significant predictors of turnover intention. Furthermore, nurses with low levels of EI, emotional labor, and social support were most likely those with high intentions of resigning from their current posts [18]. Similarly, Hong and Lee (2016) performed structural equation modelling in a study involving 211 nurses and found that EI can decrease turnover intention by regulating burnout and emotional labor rates [19].

Additionally, to quantitatively assess the predictive ability of nurse leader EI in determining clinical nurse job satisfaction, Coladonato and Manning (2017) performed a descriptive, cross-sectional study. The research was participated in by 20 nurse leaders. It included a secondary analysis of existing data from the National Database of Nursing Quality Indicators that staff members of a single hospital site in the United States filled out. Results showed that nurse leaders had average to high levels of EI. The ability to be aware of one’s own emotions and how an individual perceives oneself was found to significantly predict nurse manager performance, nurse manager leadership, level of nurse manager support offered to nurses, and staff nurse job satisfaction [20]. Similarly, Thagoe and Quarshie (2016) found that EI was significantly and positively correlated with job satisfaction in a separate cross-sectional study involving 120 nurses in Ghana [21].

In the UK, Mansel and Einion (2019) performed a qualitative study in order to explore the relationship between EI and nurse leadership. A total of 5 middle-level nurse managers were interviewed following an interpretive phenomenological method. Analysis of results showed that nurse leaders could manifest EI in four ways. First, EI pertains to sensing others (i.e., empathic leader) which includes understanding the needs of others, cultivating skills and values that staff need to care compassionately and effectively, and perceiving the lack of empathy from others. Second, EI is experiencing the affected sense of self, such as when one feels overburdened, stressed, and anxious. Third, to implement EI, strategies must promote positive feedback and teamwork. Lastly, EI pertains to the ability to read the organization’s flux, lead from a distance, and respond to poor staffing levels [22].

A way from the context of the current study, In the United States, a study examined the relationship between EI and occupational stress levels of certified registered nurse anesthetists. It is hypothesized that nurse anesthetists experience high levels of occupational stress because of their advanced levels of practice. However, it is not known whether occupational stress is associated with EI. A total of 295 nurses participated in the study. Results showed that participants had moderately high levels of EI and moderate levels of occupational stress. In addition, results showed that nurses who had a better ability to recognize and regulate their emotions had lower levels of occupational stress than those who did not [23].

In Jordan, there has been mounting, but still sparse, evidence on the role of EI within healthcare organizations. For example, al-Hamdan and colleagues (2021) explored the relationship between EI and nurse-nurse collaboration among 311 nurses working in 2 hospitals in Jordan. Results showed that nurses with high levels of EI were more likely to work effectively with each other and that nurses who worked with each other demonstrated improved levels of job satisfaction, retention, quality of patient care, efficiency, and productivity [24]. Likewise, Al-Hamdan and colleagues (2016) used a cross-sectional design in a separate study to find the relationship between EI and the job performance of Jordanian nurses working in hospital settings. Data gathered from 194 nurses working across 6 hospitals showed that nurses with high EI levels also demonstrated better performance than those with lower levels of EI. Moreover, working in medical-surgical wards, recognizing and expressing emotions and the ability to control emotions were found to be significant predictors of job performance [25].

In terms of nurse managers, Al-Hamdan and colleagues (2019a) performed a descriptive correlational study to determine the relationship between EI and conflict management styles of nurse managers in Jordan hospitals. Findings showed that nurse managers who employed integrating, compromising, and obliging styles of conflict management had higher EI, while nurses who employed dominating and avoiding styles had lower EI. The mean scores of nurse managers demonstrated moderate to high levels of EI [26]. Consistent with the correlates of EI, Al-Hamdan and colleagues (2019b) found that higher EI was significantly associated with higher intent to stay. Multivariate regression analysis showed that nurses working in private hospitals and with postgraduate degrees had significantly higher levels of EI [26].

It can be noted that although the studies mentioned earlier were performed to examine EI among nurses working in Jordan, studies still needed to be performed that examined the relationship between EI and organizational commitment. This lack of studies represents the gap in the literature that this study aims to address.

Similar to Hakami and colleagues (2020), Tuson and Ulusoy (2017) and Chegini and colleagues (2019) explored the relationships between organizational commitment and job satisfaction and extended those to include organizational justice and self-efficacy. The investigators recruited 401 nurses working in Iranian hospitals. Gathered data were analyzed using structural equation modelling. Findings confirmed the results of other studies that nurses with high levels of organizational commitment also scored highly on levels of job satisfaction. In addition, levels of organizational justice were found to affect levels of organizational commitment indirectly – if nurses were working in hospitals that were viewed as fair, objective, and honest, nurses were more likely to remain committed to those hospitals and have less intention to leave [27–29].

Lastly, some studies also explored the role of the quality of work life on the levels of organizational commitment among nurses. For example, in Iran, Hashempour and colleagues (2018) found that quality of work life was significantly and positively correlated with organizational commitment. However, the investigators did not find any significant relationship between organizational commitment and age, service record, or educational level [30]. The study was participated in by 51 nurses from a single hospital site in Iran.

## Materials and methods

The study utilized a descriptive, cross-sectional, correlational design. The research was conducted in three governmental tertiary-level hospitals in Jordan. These hospitals were chosen because of the large number of patients catered to, which reflects the healthcare demand that must be met by competent, safe, and quality nursing management. These three hospitals A, B, and C were as following: Hospital A is a comprehensive hospital in which it is composed of four hospitals: Medical Hospital, Surgical Hospital, Maternity and Paediatric Hospital, and Emergency Hospital. Moreover, it has a total capacity of 1,088 beds and a total of 1200 nurses, making it the largest governmental and referral hospital in Jordan. Second, Hospital B has a total bed capacity of 494 beds and 550 nurses. Lastly, Hospital C has a total capacity of 436 beds and 450 nurses [31].

The target population that the researcher look to generalize the current results to is all Jordanian nurses working in hospitals under the governance of the Ministry of Health. However, the accessible population is all nurses working at the three selected hospitals participating in the study.

The study recruited participants using a convenience sampling design with inclusion-exclusion criteria. Convenience sampling allowed the researcher to easily access participants with socio-demographic characteristics that fit the eligibility criteria [32]. The inclusion-exclusion criteria allowed the researcher to control extraneous variables by limiting sampling to participants with similar characteristics to each other [32]. The inclusion criteria for the study were (1) being a Jordanian registered nurse, (2) being employed in the research setting for at least 6 months, (3) providing direct patient care, (4) must possess at least a Bachelor’s degree, and (5) must have provided consent to participate in the study. In addition, nurses must be employed for at least 6 months to gain experience working in their current departments and to be able to have adequate work experience and develop a sense of the hospital’s organizational culture as well [33]. On the other hand, nurses who were in administrative or managerial roles were excluded from the study.

Sample size calculations were performed using G* Power version 3.1.9.7 based on a correlation test in order to achieve a power of 0.95, alpha = 0.05, and medium effect size of 0.3; and the numbers for the sample size calculation came from the pilot study was conducted. So, the minimum sample size required was 134. The sample size was increased to 230 participants for a possible non-response rate.

Participation in the study was completely voluntary. Participants were informed that they could only join the study if they explained. Participants were also informed that they could withdraw from the study by not completing the survey questionnaires.

The investigator provided informed consent so that potential participants could make an informed decision about whether or not to join the study. The benefits and risks of joining the study were explained. The expected role of the participants was also discussed.

The privacy and confidentiality of participants were respected at all times. Only the researcher could access all hard and online copies of participant information sheets and filled-out questionnaires. Likewise, the anonymity of participants was protected. No personally identifiable information was gathered from participants which means the researchers cannot link the responses to the participants in a way or in another. To enable the researcher to match participant information sheets with filled-out survey questionnaires, each participant was assigned a control number (a code) used across all documents. A list that the researcher can only access contains a list of the control numbers (codes). Online questionnaires were encrypted and virus protected. All collected data were stored in a password-protected computer. The computer was a virus- and malware-protected. Data protection was in line with existing data privacy laws in Jordan. Participants had not undergone any experimentation or intervention.

After obtaining ethical approval from the IRB committee at Zarqa university and the targeted hospitals, the researcher met each hospital’s director to explain the study’s purposes. Then the researcher met nurse managers in each department and informed her/him about the study’s aims. The researcher explained the study’s data collection methods as well. A list of nurses was obtained from nurse managers to identify all the potential participants after screening nurses based on inclusion-exclusion criteria. Nurses who fulfilled the study’s eligibility criteria were approached for recruitment. Finally, the researcher invited potential participants to join the study and explained the purpose, potential risks, the role of the participants, and possible benefits of joining.

Participants who agreed to join the study were asked to sign an informed consent form and were added to a WhatsApp group designed for participants. The researcher designed an electronic web-based questionnaire package that included the consent form, the objectives of the study, guidelines to fill out the questionnaires, and the instruments. Participants were given the survey questionnaires link to fill out the questionnaires. The online format allowed nurses to save and submit their responses later. However, participants were asked to complete the questionnaires within 14 days before the link expired. All questionnaires were in the English language. Finally, the researcher thanked all participants for joining the study via the WhatsApp group. Participants were also informed that they could access the study results by requesting a copy of the published materials later on or a summary in plain language.

Data collection started on August 2022 and was completed on November 2022. Out of 230 nurses invited to participate in the research, a total of 200 completed the questionnaires for a response rate of 87%.

A participant information sheet developed by the researcher was used to obtain information about the socio-demographic characteristics of the participants. Personal, professional, and work-related data were gathered using the participant information sheet, which includes age, gender, marital status, educational attainment, length of experience as a registered nurse, and length of experience working in the participating hospital site. These variables were selected based on other studies that examined the same phenomenon in the literature review [24, 34].

### Instrumentation used

The Emotional Intelligence Scale (EIS) developed by Schutte and colleagues (1998) was used to measure the emotional intelligence of nurse participants. In this instrument, emotional intelligence is expressed as a function of traits, social skills, and competencies in recognizing and managing one’s emotions and feelings [35]. The scale comprises 33 items that use a 5-point Likert scale from 1 (strongly disagree) to 5 (strongly agree). Scores are summed to obtain the overall score. There are no cut-off scores; the higher the score, the higher the level of emotional intelligence is. The lowest score is 33, while the highest is 165 [36].

The Emotional Intelligence Scale has been tested in several studies and has been shown to possess good psychometric properties [36]. The tool has demonstrated high levels of reliability with Cronbach alpha values of more than 0.90. In addition, the tool has shown significant predictive and discriminate validity (*p* < 0.001) [36]. Permission was obtained from the authors to use the survey questionnaires in this study. As well as this tool has a rational number of items that the participant can fill without being bored or leave it unfilled.

Meyer and Allen initially developed the Organizational Commitment Scale to create a tool to assess organizational commitment as a psychological state rather than as an attribute or behavior [10]. The original scale comprises 24 items, but subsequent studies that assessed its psychometric properties have reduced the scale to 18. The scale measures organizational commitment across three dimensions of affective, continuance, and normative types, and uses a 7-point Likert scale from 1 (completely disagree) to 7 (completely agree). Total scores are summed for each of the subscales and the overall scale with a possible score range of 18–126. Higher scores denote higher levels of organizational commitment. However, Magharei et al. (2021) proposed a classification system with scores of 18–42 representing low levels, 43–84 representing moderate levels, and 85–126 representing high levels of organizational commitment [37].

Meyer and Allen (1991) provided original psychometric properties of the tool, with internal consistency measured using Cronbach alpha values of 0.70–0.87 [10]. Makarem and colleagues (2013) have demonstrated the face and content validities of the tool [38]. For the 18-item modified version, Cronbach alpha values ranged from 0.69 to 0.83 for all dimensions and had acceptable face validity [38]. Permission was obtained from the authors to use the survey questionnaires in this study.

Moreover, a cover letter was sent to participants to ensure that the measurements of independent variable are not related to the measurement of dependent variable. Besides, all instruments used were valid and reliable and the items were written in standard formal language. A pilot test was done to check the clarity and simplicity of instrument items. No ambiguity was reported.

### Data pre-processing

Data cleaning was performed to resolve any missing data. The Statistical Package for Social Sciences (SPSS) version 23 was used. Some raw narrative data (such as hospital name, unit name) that were collected from participants were coded to numbers that can be easily analyzed by SPSS. The level of significance was set at 0.05; however, data were cleaned and screened for missing data, outliers by inspecting descriptive statistics and frequency distributions for all of the study variables. Descriptive statistics were performed to measure frequencies, percentages, means, and standard deviations of variables of interest. Inferential statistics were used to answer the study questions. Before applying inferential statistics, the underlying assumptions for inferential statistics were tested to ensure that the requirements were fulfilled, including the test of normality, linearity, and homogeneity of variance.

Pearson’s r was used to test whether any significant relationship exists between emotional intelligence and age and organizational commitment and age. Pearson’s r was used to test whether any significant relationship exists between emotional intelligence, length of experience, and organizational commitment and length of experience. One-way analysis of variance ANOVA was used to test whether any significant differences exist on emotional intelligence and organizational commitment based on marital status and educational attainment. Lastly, Pearson’s r was used only to test whether any significant relationships exist between emotional intelligence and organizational commitment. Lastly, causality relationship is out of the current study’s purposes.

## Results

### Socio-demographic characteristics

A total of 200 nurses participated in the study. Table 1 shows the socio-demographic and professional profiles of the participants. The mean age of participants was 33 years (M = 33 ± SD = 6.8). The sample comprised 90 (45.0%) male and 110 (55.0%) female nurses. In terms of marital status, the majority were married (*n* = 133, 66.5%), while the rest of the participants were single (*n* = 52, 26.0%), divorced (*n* = 11, 5.5%), or widowed (*n* = 4, 2.0%).

**Table 1:** Socio-demographic Characteristics (n = 200)

	Mean	SD
Age	33	6.8
	**Frequency (** ***n*** **)**	**Percentage (%)**
Gender		
Male	90	45.0
Female	110	55.0
Marital Status		
Single	52	26.0
Married	133	66.5
Divorced	11	5.5
Widowed	4	2.0
Educational Attainment		
Bachelor	140	70.0
Master’s	47	23.5
PhD	13	6.5
Years of Experience as a Registered Nurse		
< 1 year	5	2.5
1–3 years	28	14.0
4–6 years	35	17.5
> 6 years	132	66.0
Years of Experience in the Place of Work		
< 1 year	11	5.5
1–3 years	56	28.0
4–6 years	45	22.5
> 6 years	88	44.0

As for the nurses’ professional characteristics, the majority held an undergraduate degree (*n* = 140, 70.0%), while of the rest who held a postgraduate degree, 47 (23.5%) had a master’s degree, and 13 (6.5%) had held a doctoral degree. Most participants had more than 6 years of experience working as registered nurses (*n* = 132, 66.0%). The rest of the participants had 4–6 years of experience (*n* = 35, 17.5%), 1–3 years of experience (*n* = 28, 14.0%), and less than 1 year of experience (*n* = 5, 2.5%). Similarly, most participants had more than 6 years of experience working in their current department or place of work (*n* = 88, 44.0%). The rest of the participants had 4–6 years of experience (*n* = 45, 22.5%), 1–3 years of experience (*n* = 56, 28.0%), and less than 1 year of experience (*n* = 11, 5.5%).

Based on the score interpretation guide provided for by the authors, mean overall score of participants was (M = 122.3 ± SD = 14.0) which indicates a high level of emotional intelligence. Whereas the mean overall scores of participants showed a moderate level of organizational commitment (M = 81.6 ± SD = 15.7).

One-way ANOVA results as shown in Table 2 indicated significant difference in emotional intelligence based on marital status (F = 2.93, P < 0.5). Post-hoc analysis was performed using least significant difference (LSD). The results showed that widowed nurses had significant higher emotional intelligence scores than single or married nurses (p < 0.05). Similarly, for educational attainment, nurses with doctorate degrees had significant higher emotional intelligence scores than nurses with a bachelor or master’s degree (p < 0.05). On the other hand, no significant difference in emotional intelligence was found based on years in the area of work (F = 1.10, P = 0.35), or years in the current hospital (F = 0.51, P = 0.68).

**Table 2:** Differences in emotional intelligence and organizational commitment based on socio-demographic characteristics

Variable	Category	EI	A	C	N	Overall OC
Age	*r*	0.13	0.004	-0.07	0.06	-0.007
	P-value	0.07	0.96	0.31	0.43	0.93
Gender	*t*	-1.13	1.16	0.98	1.09	1.28
	P-value	0.08	0.19	0.09	0.006*	0.04*
Marital Status	F	2.93	1.35	2.24	2.42	2.24
	P-value	0.04*	0.26	0.09	0.07	0.08
Educational Attainment	F	4.91	0.21	5.56	1.71	2.54
	P-value	0.008*	0.81	0.003*	0.18	0.08
Years in the Area of Work	F	1.10	0.69	0.79	2.09	1.40
	P-value	0.35	0.56	0.50	0.10	0.24
Years in the Current Hospital	F	0.51	0.90	0.19	2.59	1.22
	P-value	0.68	0.44	0.90	0.054	0.30

### Emotional Intelligence and Organizational Commitment

Pearson’s *r*-test results as shown in Table 3 summarize the relationship between the two primary variables of interest. The results showed that overall emotional intelligence had a significant, moderately strong, positive relationship with overall organizational commitment (*r* = 0.53, *p* < 0.01). This meant that nurses with a high emotional intelligence score exhibited high organizational commitment scores. Similar results were found on the relationship between emotional intelligence and affective domain (*r* = 0.40, *p* < 0.01), emotional intelligence and continuance domain (*r* = 0.48, *p* < 0.01), and emotional intelligence and normative domain (*r* = 0.45, *p* < 0.01).

**Table 3:** Relationship between emotional intelligence and organizational commitment

Variable	Category	A	C	N	Overall OC
EI	*r*	0.40	0.48	0.45	0.53
	P-value	0.000*	0.000*	0.000*	0.000*

Legend: EI – emotional intelligence, A – affective domain, C – continuance domain, N – normative domain, OC – organizational commitment, * - significant at *p* < 0.01

## Discussion

The results of this study showed that Jordanian nurses had high levels of emotional intelligence. This meant that nurses had the ability not only to be aware of their prevailing emotions but also had the capacity to take advantage of those emotions and regulate them to achieve positive goals and outcomes. The findings of the current study are in line with results of the series of research by Al-Hamdan and colleagues (2016, 2019a, 2021) who also found high levels of emotional intelligence in nurses working in Jordanian hospitals. Specifically, their study which focused on nurse managers showed moderate to high levels of emotional intelligence [24–26].

While results suggested that Jordanian nurses are emotionally intelligent, the findings may have depended on the predominant contextual factors of the healthcare system and the wider society at the time of data collection [5]. For instance, the participating hospitals might have had support systems in place to ensure that nurses can access and avail of services that will help them to become resilient and responsive to the ever-growing demands of the profession and the growing Jordanian population. Consequently, when support systems are no longer available, or when wider societal factors (e.g., economic, financial, political and social contexts) endanger personal circumstances of nurses, then the level of emotional intelligence might be negatively influenced [15]. Hence, the challenge is not so much in achieving higher levels of emotional intelligence (as current data already hinted at satisfactory levels) but more so in ensuring that already high levels of emotional intelligence are nurtured and sustained – this can be in the way of audits, continuous quality improvement efforts, and quality assurance projects. Moreover, future research should be conceptualized and carried out to widen the database involving Jordanian nurses as investigations in this topic remain sparse.

Furthermore, The results of the study showed that participants had moderate levels of organizational commitment. Moderate levels can also be seen in the affective, continuance and normative domains. Specifically, these results meant that (1) on the affective domain, nurses were emotionally attached to their organization, having found positive feelings and emotions linked with their work experience, (2) on the continuance domain, nurses found that they would be disadvantaged if they were to leave their organization, and (3) in the normative domain, nurses felt that staying with their organization was the morally right thing to do [9]. Hence, nurse participants had less desire to leave their organization and more willingness to remain because of the degree of attachment or bond they had [39].)

The findings of the study were similar to those by Hakami and colleagues (2020) in Saudi Arabia and Karami and colleagues (2017) in Iran who found moderate levels of organizational commitment among nurse participants [28, 40]. However, the results of the study were lower compared to those of Albasal and colleagues (2022) who measured high levels of organizational commitment among a different sample of Jordanian nurses [41]. The variation in scores were most likely due to contextual factors unique to each working environment since nurses with high levels of organizational commitment were found to work with hospitals that demonstrate better service delivery, processes and outcomes [42]. Nevertheless, lower scores are suggestive of rooms for improvement, allowing nurse managers and hospital administrators to plan interventions that can develop and nurture the level of organizational commitment among staff nurses [43]. Ensuring that nurses are committed to stay in the organization does not only support the sustenance of the nursing workforce in an environment of nursing shortages and poor skill mix but more so guarantees that nurses are satisfied with their jobs, roles and daily responsibilities [44, 45].

The study’s results showed that emotional intelligence and organizational commitment had a significant,, positive relationship with each other. This meant that nurses who could manage and regulate their emotions approprietly were more likely to be attached and bonded to their organization. In other words, nurses who posses higher degrees of emotional intelligence tend to be more commited to their organizations, build robust working relationships, are tolerant when encountering emotional labour or emotional distress and can handle the odd feelings without losing their tempers. Beside, they can deal work-family conflicts in a befitting manner. Actually, the possible reasons for this positive relationship are that emotionally intelligent staff who know themselves well and can manage their own feelings and those of others are seemed to be more engaged and committed to their affiliated organizations. Therefore, nurse managers should take the opportunity to develop the emotional intelligence of nurses, providing them not only with services to increase their knowledge and develop their skills in managing their emotions but also with the support to have the necessary time to reflect on how their emotions might be used to the advantage of their patients, families and the more comprehensive organization [11]. It is important here to mention that this relationship between emotional intelligence and organizational commitment does not guarrantee a causality relationship between the two variables (i.e. emotional intelligence does not cause organizational commitment).

Moreover, there should be efforts to improve the quality of the nursing work environment within hospitals and other healthcare settings because both emotional intelligence and organizational commitment are context-based as results of the study have shown – better work conditions, safe infrastructure and equipment, adequate resources and supplies, and efficient processes and services increase the extent by which nurses can effectively carry out their jobs and improve their perceptions towards their peers, managers, patients and other service users [18, 46].

As to the researcher’s knowledge, this is the first study that demonstrated the link between emotional intelligence and organizational commitment among nurses working in governmental hospitals in Jordan. Therefore, future research should generate more data in exploring these variables. Moreover, future research should look into the factors that strengthen or weaken the relationship between emotional intelligence and organizational commitment, especially since emotional intelligence seems to be linked with organizational commitment via the latter’s affective domain. Whether there is a link between the two variables or an association was only found because the similarity of theoretical concepts between emotional intelligence and the affective domain needs to be further explored [47].

Significant results on emotional intelligence and organizational commitment were only found on gender, marital status and educational attainment, with male, widowed and better educated nurses demonstrating higher scores than female, single and nurses with undergraduate degrees. With regard to gender, it was interesting to find that male participants had significantly higher levels of organizational commitment than their female counterparts. This result was contrary to studies indicating that female nurses have intrinsically better grasp, awareness and regulation of their emotions, and tend to adhere better to organizational values and loyalties [48, 49]. In contrast, previous studies have found lower levels of organizatioal commitment among male nurses because of perceived role conflict (i.e., “nursing is a woman’s job”) and desire for a higher paying job [50, 51]. Unfortunately, there is not enough evidence in the data to support any inferences why male nurses had significantly higher levels of organizational commitment than female nurses. This contrasting result, and a more in-depth investigation of the role of gender in organizational commitment of nurses, can be explored in future research.

On the other hand, postgraduate education provides a better layer of maturity and understanding to nurses, therefore equipping them with the necessary knowledge and skills to become more responsive, resilient and skillful in addressing professional demands, issues and challenges [52]. There might also be a probability that the postgraduate degree was a product of an educational opportunity from their employers, thus signalling employer commitment to develop staff nurses personally and professionally. On the other hand, there was not sufficient data from the results to explain why widowed nurses might have better scores on emotional intelligence compared to single and married nurses but it can be surmised that going through a stressful and emotionally-exhaustive experience of losing a spouse might lend better emotional maturity to an individual [53]. However, it does not mean that nurses should be widowed to become more emotionally intelligent – nurse managers should implement interventions such as workshops, one-on-one feedback sessions and individualized follow-ups that can improve emotional intelligence and ensure that effects are sustainable and consistent over time [11].

### Implications

Nurses with high levels of emotional intelligence have the capacity not only to become more competent and responsive in providing safe and quality care to patients and their families but also can harness their awareness of their emotions to cope with the increasing pressures and demands of their job and the healthcare delivery system as a whole. For example, being emotionally intelligent meant that nurses do not become overwhelmed by negative feelings brought about by stress and fatigue but instead find ways to manage and deal with such emotions. In addition, nurses with high levels of organizational commitment meant that their attachment or bond to their organizations was strong enough to persuade them to stay in their current jobs and roles. Being committed to one’s organization implies loyalty and a willingness to remain despite issues, problems, and challenges. Moreover, when nurses are committed to their roles and responsibilities, patients, families, and other service users are ensured to receive dedicated, devoted care up to quality standards.

With the ongoing international shortage of nurses, nurses must remain committed to their organizations. Having nurses who have high levels of organizational commitment ensures a sustainable workforce that retains both junior and experienced nurses. Similarly, having nurses who have high levels of emotional intelligence promotes a spirit of resilience and strength. It cannot be denied that healthcare systems are under constant pressure to provide high levels of quality care despite the incessant demands of a growing population. Thus, emotional intelligence is a significant ingredient that can support nurses in dealing with such pressures. Therefore, healthcare organizations’ policies should focus on organizational commitment and emotional intelligence.

### Recommendations

Evaluating the levels of emotional intelligence and organizational commitment of nurses should be included in hospital audits and quality improvement efforts. Auditing ensures that interventions to improve emotional intelligence and organizational commitment are routinely implemented and performed according to content and standards. Quality improvement efforts ensure that problems and issues identified in implementing interventions are identified, resolved, and prevented from happening in the future. Moreover, recognizing emotional intelligence and organizational commitment as priority organizational outcomes should be translated to standard measurement levels among nurses to ensure that interventional effects are consistent and sustainable.

Practically, interventions aiming to improve organizational commitment and emotional intelligence should be formulated and implemented. These can be in the form of lectures and seminars, first to increase knowledge and awareness among nurses in the significance of these variables, and then followed by training sessions to teach nurses the skills to become emotionally aware, resilient and flexible.

Moreover, since more experienced and mature nurses demonstrated significantly higher levels of emotional intelligence and organizational commitment than junior nurses, nurse managers should ensure that systems are in place to support junior nurses’ personal and professional development in the workplace. For instance, there should be opportunities to pursue postgraduate education for nurses who only have undergraduate degrees. Such support systems should be consistent, accessible, available, and sustainable over some time.

Besides, nurse researchers should take the lead in conceptualizing and developing future studies on the emotional intelligence and organizational commitment of nurses working in Jordanian healthcare settings. Topics that merit further investigation are (1) individual- and organizational-level factors that influence the levels of emotional intelligence and organizational commitment and (2) the effectiveness of interventions that can improve levels of emotional intelligence and organizational commitment.

### Limitations and conclusion

Despite the strength points of the current study, it has some limitations. First, the cross-sectional nature of the research design meant that variables were only measured at a snapshot of time, thus making it difficult to make inferences regarding the state of emotional intelligence and organizational commitment over time. When organizational contexts and individual or personal circumstances change, the study’s results may no longer take hold. Second, the study relied on self-report, which meant that results were exposed to possible risks and inaccuracies from recall. Third, emotional intelligence and organizational commitment were measured based on the point of view of the individual nurse and not on that of their peers, managers, or employers. Third, the study was limited to nurses working in only three governmental hospitals and did not include nurses working in other healthcare settings such as private hospitals, community clinics, and primary care centers.

Moreover, the research sites were chosen based only on access and offered services but were not systematically and randomly chosen among other governmental hospitals in Jordan. Thus, the study results have limited generalizability and can be applied only to nurses and hospitals with similar socio-demographic and organizational characteristics as that of the research settings and study participants.

Emotional intelligence and organizational commitment have a significant positive relationship with each other, therefore offering support to the planning and implementation of interventions that will improve the way nurses manage their emotions and the extent to which nurses are attached to their job roles and responsibilities of providing safe and quality patient care.

## Data Availability

All data generated or analyzed during this study are included in this published article.
